# Processing and Properties of Tungsten-Steel Composites and FGMs Prepared by Spark Plasma Sintering

**DOI:** 10.3390/ma15249037

**Published:** 2022-12-17

**Authors:** Jiří Matějíček, Radek Mušálek, Zdeněk Dlabáček, Veronika Klevarová, Lenka Kocmanová

**Affiliations:** 1 Institute of Plasma Physics of the Czech Academy of Sciences, 182 00 Prague, Czech Republic; 2 Faculty of Mathermatics and Physics, Charles University, 116 36 Prague, Czech Republic; 3 Faculty of Nuclear Sciences and Physical Engineering, Czech Technical University in Prague, 166 36 Prague, Czech Republic

**Keywords:** plasma facing components, tungsten, steel, spark plasma sintering, composites, FGMs

## Abstract

Tungsten is the prime candidate material for the plasma-facing components of fusion reactors. For the joining of tungsten armor to the cooling system or support structure, composites or graded interlayers can be used to reduce the stress concentration at the interface. These interlayers can be produced by several technologies. Among these, spark plasma sintering appears advantageous because of its ability to fabricate fully dense parts at lower temperatures and in a shorter time than traditional powder metallurgy techniques, thanks to the concurrent application of temperature, pressure, and electrical current. In this work, spark plasma sintering of tungsten-steel composites and functionally graded layers (FGMs) was investigated. As a first step, pure tungsten and steel powders of different sizes were sintered at a range of temperatures to find a suitable temperature window for fully dense compacts. Characterization of the sintered compacts included structure (by SEM); porosity (by the Archimedean method and image analysis); thermal diffusivity (by the flash method) and mechanical properties (microhardness and flexural strength). Compacts with practically full density and fine grains were obtained; while the temperature needed to achieve full sintering decreased with decreasing powder size (down to about 1500 °C for the 0.4 μm powder). For fully sintered compacts, the hardness and thermal diffusivity increased with decreasing powder size. Composites with selected tungsten/steel ratios were produced at several conditions and characterized. At temperatures of 1100 °C or above, intermetallic formation was observed in the composites; nevertheless, without a detrimental effect on the mechanical strength. Finally, the formation of graded layers and tungsten-steel joints in various configurations was demonstrated.

## 1. Introduction

Plasma-facing components in future fusion reactors will be subjected to extremely harsh conditions, namely high particle and heat fluxes from the hot plasma [1]. Such a demanding environment poses stringent requirements on the applied materials, which can be fulfilled only by very few of them. Tungsten is the prime candidate for the plasma facing armor, namely thanks to its refractory nature (high melting point and high strength at elevated temperatures), high sputtering threshold, good thermal conductivity, low tritium retention, etc. [2,3]. However, it has serious limitations due to its mechanical properties (brittleness at low temperatures, propensity to recrystallization at higher temperatures, poor machinability) [2]. Therefore, it can be used only as an armor, without a structural function. The armor needs to be joined to a cooling or load-bearing construction system. For ITER, the largest tokamak currently under construction, the cooling system of the divertor will be made of copper-based material [4]. For DEMO, the future demonstration power plant, reduced-activation ferritic-martensitic steel is foreseen as the main structural material, primarily for the first wall/blanket system [5,6,7]. For both material combinations—W/Cu and W/steel—there is a large mismatch of thermal expansion coefficients (CTEs), which leads to stress concentration upon thermal excursions. To alleviate the stress concentration at the joint, replacement of a sharp interface by a gradual transition may be beneficial [5,6,7]. According to [6], at least 1 mm thickness of the graded layer is needed for proper redistribution of the stresses. In this case, the role of the so-called functionally graded materials (FGMs) is to provide a gradual transition of CTEs, whereas in other applications, such as aerospace, metal–ceramic FGMs are often used, taking advantage of the mechanical strength of metals and the thermal and corrosion resistance of ceramics [8,9]. For the FGM formation in fusion applications, several techniques have been explored. These include, for example, coating techniques (plasma spraying, laser cladding, physical vapor deposition, electrodeposition) and powder metallurgy techniques (hot isostatic pressing, hot pressing, spark plasma sintering, resistance sintering under ultra-high pressure). A preliminary assessment of their advantages and drawbacks was provided, e.g., in [10,11]. From the latter group, spark plasma sintering holds the advantage of achieving fully dense products at lower temperatures and shorter times than conventional sintering techniques [12,13]. The limited thermal exposure induces only moderate grain growth and reduces the likelihood of intermetallic formation, etc., which could be beneficial for the properties and performance of the consolidated materials [14].

For the most exposed components, several mm of tungsten are needed for the armor, to ensure adequate thermal protection of the underlying materials, as well as a sufficient lifetime of the armor itself, which will undergo erosion by the plasma particles [15]. Such thickness can be achieved only by bulk fabrication techniques, while the coating techniques are applicable only to less exposed components.

This study is dedicated to the processing of tungsten-steel composites and FGMs by spark plasma sintering and their characterization. The aims of this work were as follows:

- Find optimal sintering conditions for the pure tungsten and steel materials and their composites;
- Perform basic characterization of the relevant properties;
- Demonstrate the capability of the SPS technique to form FGMs in various configurations as well as joints with bulk counterparts.

First, process optimization for pure tungsten and steel products are presented together with their key characteristics. The second part will focus on the formation and characterization of composites, FGMs, and joints.

## 2. Materials and Methods

Pure commercial tungsten powders of several nominal sizes (as designated by the manufacturers), −20 μm (Osram Sylvania, Towanda, PA, USA), ~4 μm, ~2 μm, ~0.7 μm and ~0.4 μm (Global Tungsten and Powders, Bruntál, Czech Republic) and P91 steel powder (−20 μm, Karlsruhe Institute of Technology, Karlsruhe, Germany) were used. The sintering was performed in an SPS 10-4 machine (Thermal Technology, Santa Rosa, CA, USA) at 60–80 MPa pressure for 2 min at the maximum temperature, using graphite die and punches lined with a graphite foil and an inert atmosphere. Sintering temperatures were varied and will be mentioned below in specific cases. The temperatures were measured by a pyrometer pointing at a narrow hole in the die, about 5 mm from the sample surface (standard setup of the device for high-temperature materials). Sintered compacts of ~19 mm diameter and ~3 mm thickness were prepared.

Structural observations were performed in an EVO MA-15 scanning electron microscope (SEM; Carl Zeiss SMT, Oberkochen, Germany) on polished cross-sections. Porosity was determined by the Archimedean method (AM) with water immersion and, alternatively, by image analysis (IA) of the SEM images of polished cross-sections [16]. For the latter, ImageJ software (v. 1.44, National Institutes of Health, Bethesda, MD, USA) was used, applying the area fraction metric after suitable thresholding. Five images were taken from each sample. Thermal diffusivity was determined by the flash method [17,18], using FL-3000 (Anter Corp., Pittsburgh, PA, USA) and LFA 1000 (Linseis, Selb, Germany) instruments. Microhardness measurements [19] were performed on polished cross-sections using a Nexus 4504 (Innovatest, Maastricht, The Netherlands) instrument with a Vickers indenter, 3 N load and 10 s loading time, taking an average of 12 indentations positioned in the central region of the sample. The flexural strength of selected composites was determined by three-point bending on 19 × 4 × 3 mm samples using an Instron 1362 universal testing machine with an 8800 electronic control system (Instron, High Wycombe, UK). Fractographic analysis of the broken samples was carried out by SEM in the areas loaded in tension mode.

## 3. Results

### 3.1. Pure Tungsten

**Figure 1:**
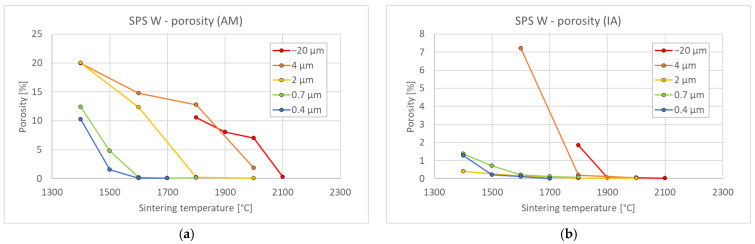
Effect of sintering temperature on porosity for tungsten of different powder sizes; (**a**) results from Archimedean method (AM), entire sample; (**b**) image analysis (IA), dense central region.

**Figure 2:**
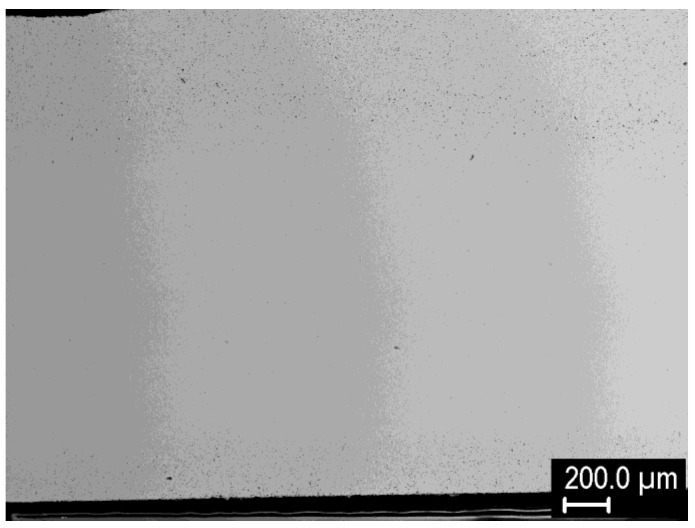
Cross-section of tungsten compact sintered at 1900 °C from the −20 μm powder.

To find suitable conditions for proper sintering, a range of temperatures was used for each powder. Figure 1 shows the porosity dependence on sintering temperature. Expectedly, the porosity decreases with increasing temperature. In addition, for finer powders, the temperature needed to achieve nearly full density decreased from ~2100 °C for the −20 μm powder to ~1500 °C for the 0.4 μm powder. This agrees reasonably with the study by Autissier et al., where densities above 95% were achieved at 1900 °C for 5–10 μm powder [20]. Cross-section observation of the non-fully sintered compacts (Figure 2) revealed that the porosity is concentrated close to the sample surfaces, while the central region is much denser. This was confirmed by image analysis in the central regions of the respective samples (Figure 1b) showing significantly lower porosity values. Nevertheless, sintering conditions leading to nearly full density are important, and the corresponding temperature range was determined from the Archimedean porosity data.

Besides the sintering temperature, the applied pressure and dwell time are also important parameters. For most of the experiments, a maximum pressure (limited by the nominal strength of the graphite die) of 80 MPa was used. When a slightly lower pressure of 60 MPa was used for the sake of longevity of the graphite die, similar porosities were observed (Table 1). Therefore, in this range, the applied pressure is not the critical parameter. Regarding the sintering time, the densification progress was monitored in real time during the experiments (by displacement of the punches), and it was found that 2 min were sufficient to achieve a steady state. Such short processing times present a significant advantage of the SPS technique over traditional sintering techniques.

**Figure 3:**
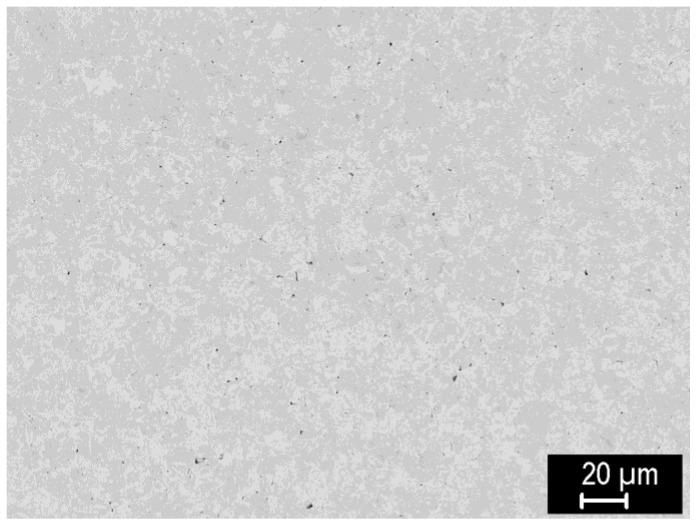
Microstructure of tungsten compact sintered at 2000 °C from the −20 μm powder.

Figure 3 shows a representative microstructure of a nearly fully sintered −20 μm W powder, prepared at 2000 °C. Grains typically with several μm in size are observed, with a slight orientational contrast apparent in back-scattered electron observation mode, which is typical for polycrystalline tungsten. Minor residual porosity can be observed as well. Similar microstructures were observed for fully sintered compacts from other powder sizes as well.

**Figure 4:**
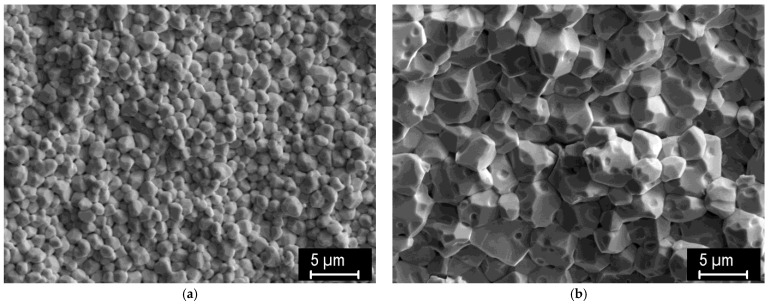
Fracture surfaces of samples sintered from the 0.4 μm powder; (**a**) at 1400 °C, (**b**) 1700 °C.

To get a clearer picture of typical grain sizes, surfaces of intentionally fractured samples were also observed. Figure 4 shows representative fracture surfaces of the ~0.4 μm powder sintered at 1400 and 1700 °C. Grain sizes between 0.5 and 1 μm and between 2 and 3 μm are observed at 1400 and 1700 °C, respectively. A similarly moderate increase in grain size with the sintering temperature was found for the other powders. This indicates that grain growth is rather limited, thanks to the relatively short processing times. In the study by Ren et al., nanometric W powder (~50 nm) was used as a starting material for pressureless sintering, still reaching similar grain sizes at conditions leading to ~full sintering [21].

**Figure 5:**
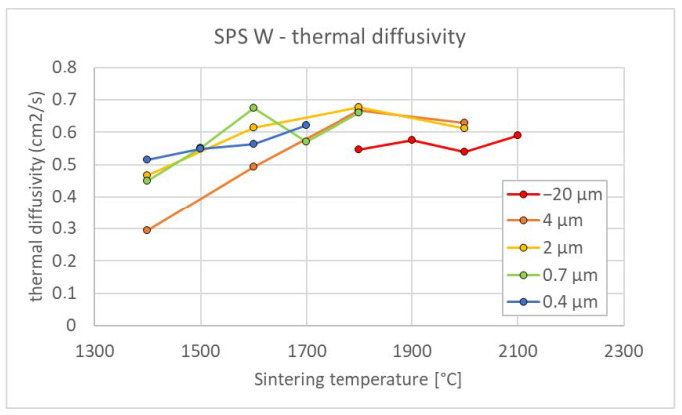
Influence of sintering temperature and powder size on the thermal diffusivity of tungsten compacts (measured at 100 °C). Typical coefficient of variation of the thermal diffusivity measurements is about 5% [17].

Figure 5 shows the dependence of thermal diffusivity on sintering temperature. A trend of increasing diffusivity with increasing sintering temperature is observed, as could be expected by an improved densification. In addition, the following observations can be made. For a moderate variation in temperature—and therefore porosity—diffusivity varies only a little. This is in contrast to plasma sprayed coatings, for example, where even a relatively small volume of (largely anisotropic) porosity can reduce the diffusivity significantly [22]. Therefore, from the heat transfer point of view—being important for plasma facing components—a small departure from full sintering is not critical. However, even a small amount of porosity can considerably affect the interaction with plasma, as shown in [23]. A second observation from Figure 5 is that, at the same sintering temperature, compacts from finer powders generally exhibit slightly higher diffusivity (due to lower porosity). The results for well-sintered compacts agree with the literature values for conventional bulk tungsten (0.612 cm^2^/s at 100 °C [24]).

Microhardness values of selected samples are presented in Table 2. For the −20 μm powder, an increase of hardness with sintering temperature is observed—an inverse correlation with porosity. For the finer powders, however, the similar or even lower temperatures correspond already to the regime of full sintering, and the sintering temperatures do not seem to have a strong effect on hardness. Decreasing the powder size leads to a notable increase in hardness, as already observed in [25]. Although yield strength was not directly measured, its correlation with hardness was demonstrated on a range of metallic materials (e.g., [26,27]). Therefore, the hardness trend can also serve as a qualitative indication of the trend in yield strength.

### 3.2. Pure Steel

**Figure 6:**
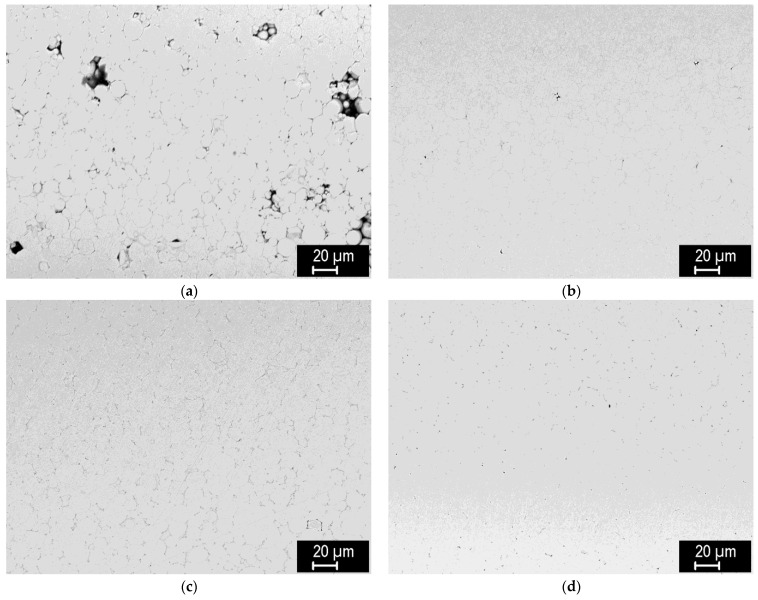
Microstructures of the steel compacts (central regions) sintered at (**a**) 800 °C, (**b**) 900 °C, (**c**) 1000 °C, (**d**) 1100 °C.

**Figure 7:**
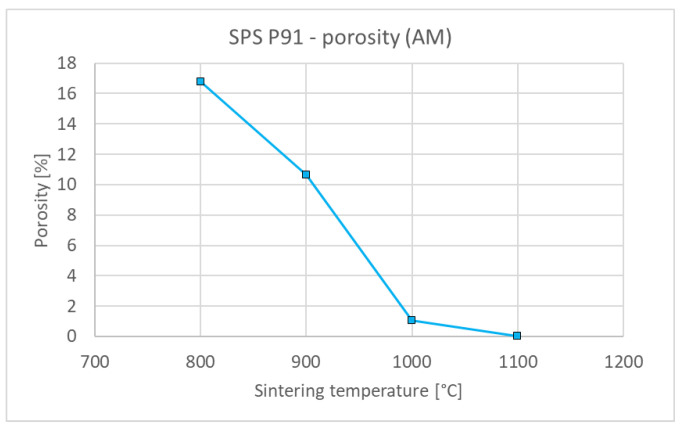
Influence of sintering temperature on the porosity of steel compacts.

**Figure 8:**
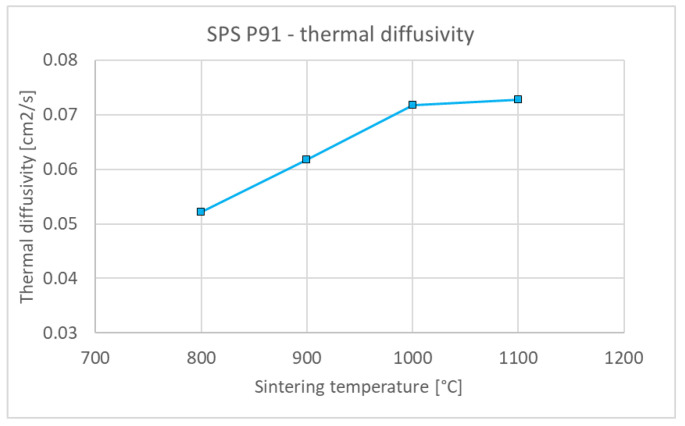
Influence of sintering temperature on the thermal diffusivity of steel compacts (at 100 °C).

Figure 6 shows representative microstructures of the steel compacts sintered at 800–1100 °C. Significant densification can be seen already at 800 °C, while the boundaries of the original particles are discernible up to 1000 °C. At 1100 °C, steel is practically fully sintered, with only a minute amount of residual porosity. Similarly as with tungsten, the porosity monotonously decreases with the sintering temperature (Figure 7) and, correspondingly, thermal diffusivity increases (Figure 8). It is worth noting that the thermal diffusivity of steel is roughly one order of magnitude lower than that of tungsten. This bears important consequences for the design and performance of plasma facing components made with this material combination—in contrast to those made of tungsten and copper [28] whose thermal conductivity is more than twice that of tungsten. Since the steel sinters fully at much lower temperatures than tungsten, no extensive variation of the conditions, as performed for tungsten, was deemed necessary.

### 3.3. Tungsten-Steel Composites

**Figure 9:**
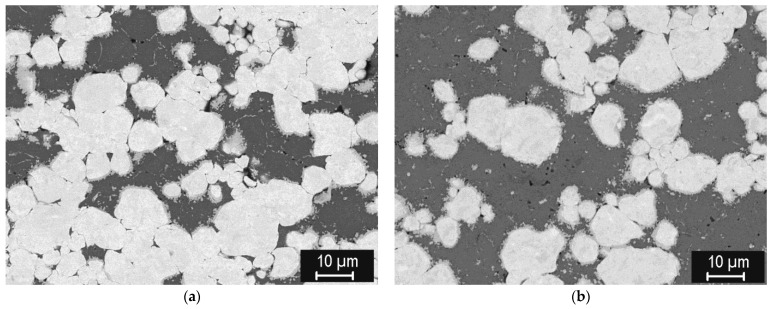

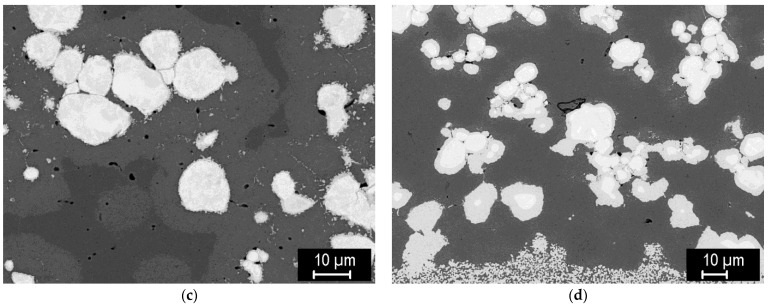
Microstructures of tungsten-steel composites sintered at 1100 °C. (**a**) 75% W, (**b**) 50% W, (**c**) 25% W, (**d**) 25% W, near the sample surface.

For the formation of tungsten-steel composites, a range of sintering conditions was also explored, albeit in a narrower range. Figure 9 shows the representative microstructures of 75/25, 50/50, and 25/75 mixtures of tungsten and steel powders, both with −20 μm size, sintered at 1100 °C.

The formation of well-sintered, dense composites at this temperature can be seen. In addition, a thin layer of intermetallic (medium-grey phase) was formed on the tungsten/steel boundaries, due to mutual interdiffusion at the elevated temperature. Strings of fine intermetallic particles can occasionally be seen in the steel phase, likely at the former steel particle boundaries. In the 25% W composite (Figure 9c), thick diffusion regions in the steel phase, without sharp boundaries, can be seen, indicating W-enriched areas around the W particles. In the 50% W and 75% W composites, such regions extend over the entire steel phase, due to shorter distances between the W particles. Figure 9d shows a microstructure of the same 25% W composite, closer to the sample surface. Here, the intermetallic layer is significantly thicker, illustrating that the interdiffusion may be affected by local variations in the current density and temperature during sintering.

**Figure 10:**
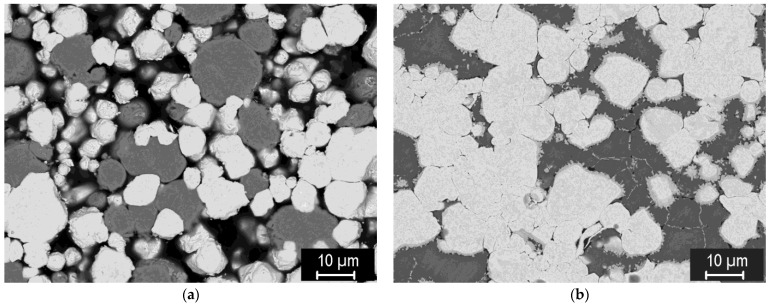

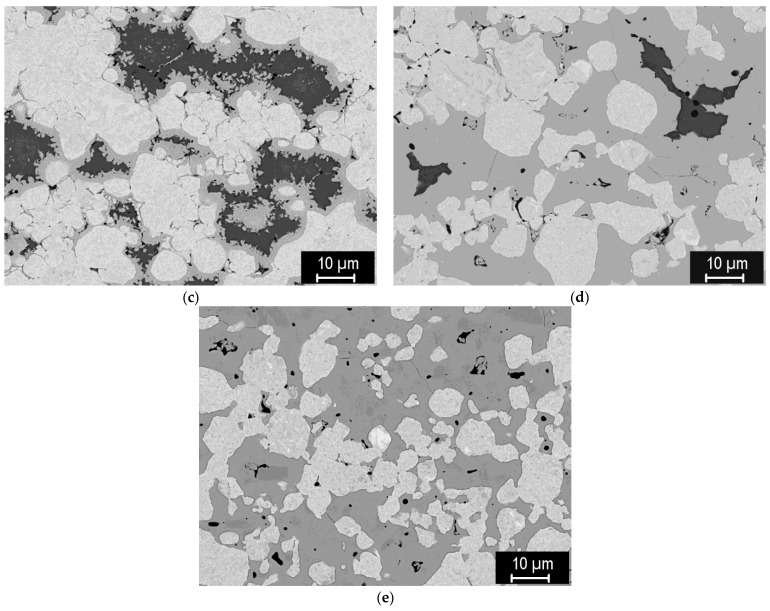
Microstructures of 75% W—25% steel composites sintered at various temperatures: (**a**) 1000 °C, (**b**) 1100 °C, (**c**) 1200 °C, (**d**) 1300 °C, (**e**) 1400 °C.

**Figure 11:**
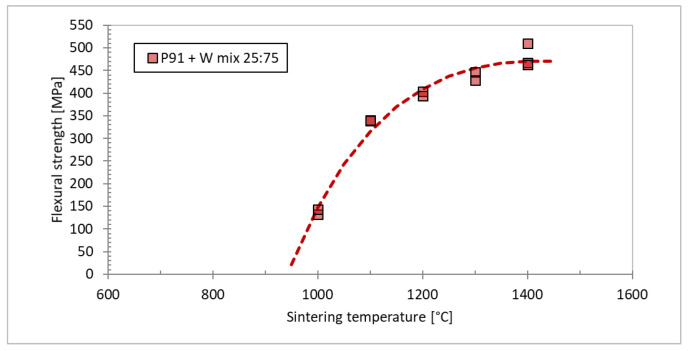
Influence of sintering temperature on the flexural strength of tungsten-steel composites.

To study the effect of the sintering temperature on the degree of mutual bonding and the intermetallic formation, sintering temperatures in the range of 1000 °C to 1400 °C were used, see Figure 10. One can see that the composites formed at 1100 °C or above are dense, while the intermetallic content progressively increases with the temperature, until at 1400 °C it represents the dominant phase. Cracks can be seen in the intermetallic (Figure 10d,e), spanning the entire thickness between W particles, testifying to its brittle nature. At 1000 °C, its formation can be avoided; however, such conditions lead to only partial sintering with rather high porosity. In [29], the properties of the individual phases in these composites were investigated. The intermetallic phase was identified by X-ray diffraction as having a Fe_7_W_6_ structure. It was found to have significantly higher hardness and yield strength than tungsten, and a thermal conductivity slightly lower than P91 steel. Here, the effects on the overall mechanical properties will be presented. Figure 11 shows the flexural strength of the composites from Figure 10, determined by 3-point bending. A gradual increase of flexural strength with the sintering temperature can be seen. This can be attributed to the improved bonding of the particles. The intermetallic content (expectedly) increases with temperature; one can conclude that—despite its brittle nature—it does not undermine the overall strength of the composite.

**Figure 12:**
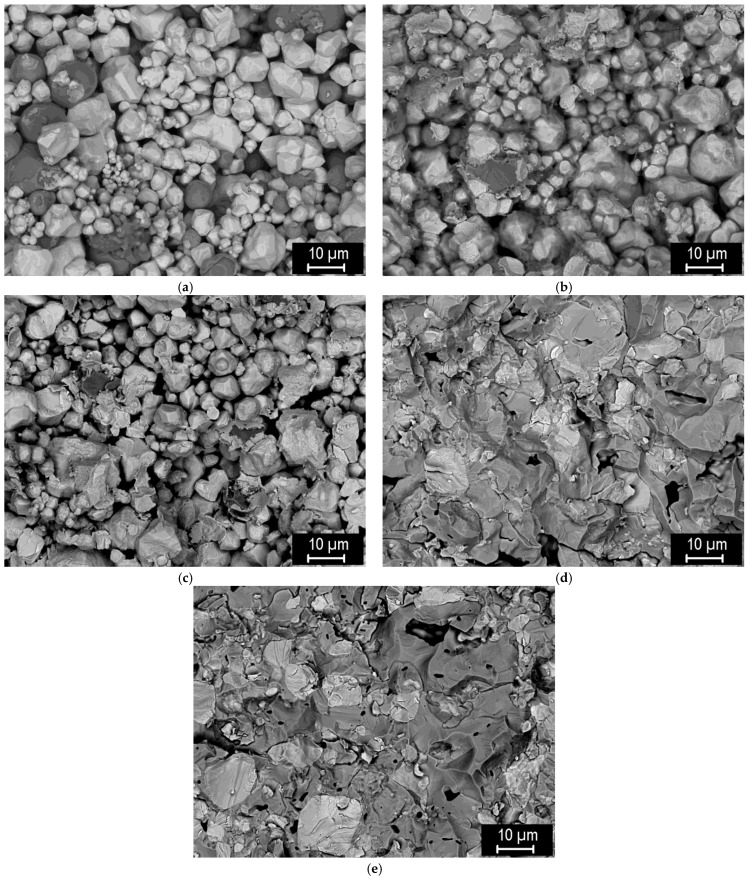
Fracture surfaces of 75% W—25% steel composites sintered at various temperatures: (**a**) 1000 °C, (**b**) 1100 °C, (**c**) 1200 °C, (**d**) 1300 °C, (**e**) 1400 °C.

Figure 12 shows details of the fracture surfaces of the composites after the 3-point bending tests. In all cases, fracture surfaces correspond to the brittle nature of the composites as seen during the bending test. Highly porous sample sintered at 1000 °C displayed easy debonding of loosely interconnected grains (Figure 12a). On the contrary, for the samples sintered at 1300 °C and 1400 °C with saturated flexural strength, transgranular failure was dominant (Figure 12d,e). For both the tungsten and the intermetallic phase, a cleavage fracture was typical. Secondary cracks were also observed, most of which nevertheless originated from cracks originally present within the intermetallic phase as observed on the cross-sections (Figure 10d,e). However, the bonding of tungsten and the surrounding intermetallic phase was excellent. Samples with intermediate sintering temperatures of 1100 °C and 1200 °C (Figure 12c,d) showed a mixture of intergranular debonding and a brittle intragranular fracture within the evolving intermetallic phase corresponding to their intermediate flexural strength.

Similarly to flexural strength, an increase in elastic moduli with the sintering temperature was observed, as determined by resonant ultrasound spectroscopy [30].

### 3.4. FGMs and Joints

**Figure 13:**
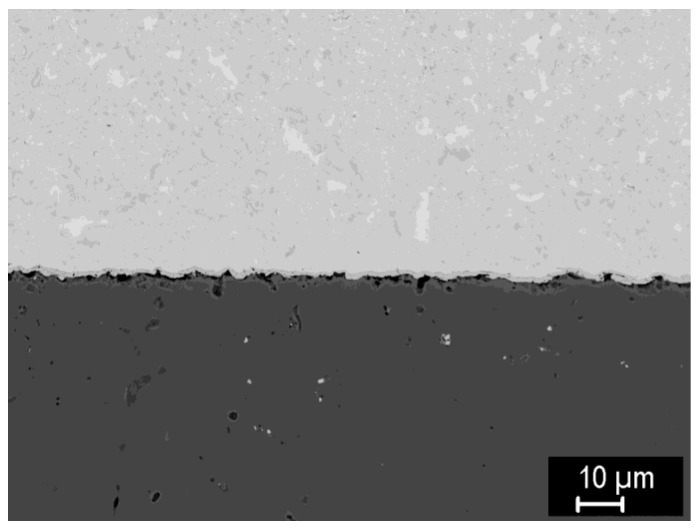
Detail of a direct joint of pure steel (**bottom**) and pure tungsten (**top**) pellets joined at 1100 °C.

**Figure 14:**
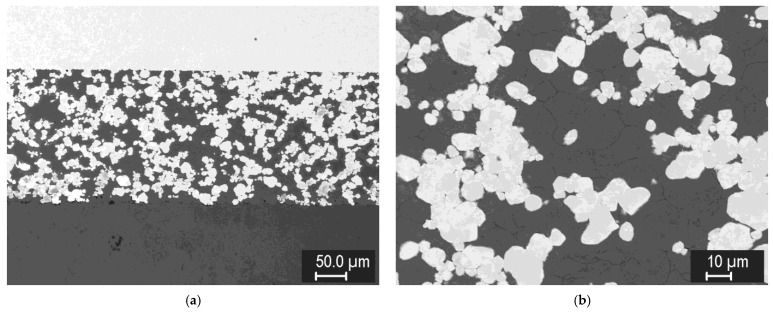

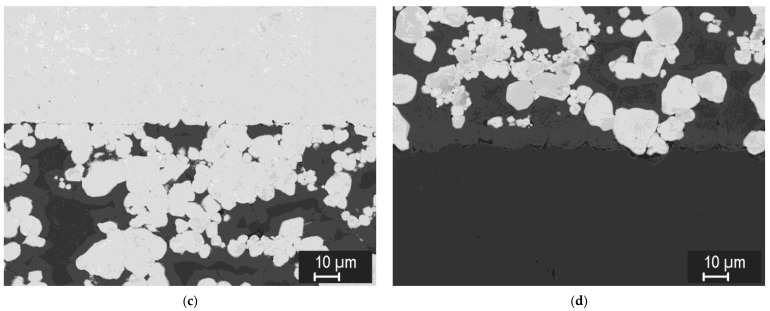
Tungsten-steel joint with a 50/50 mixed interlayer, joined at 1100 °C. (**a**) overview; (**b**) detail of the interlayer; (**c**) detail of the tungsten-composite interface; (**d**) detail of the composite-steel interface.

Since the composites would ultimately be used as interlayers between bulk tungsten and bulk steel, the formation of FGMs and joints was also explored. Figure 13 shows a direct joint of pre-sintered pellets of pure W and pure steel, joined in SPS at 1100 °C. A rather intimate contact along the interface is observed, with a thin layer (≤1 μm) of intermetallic and some porosity on the steel side. As an alternative to direct joints with a sharp interface, the introduction of a thin interlayer between the steel and tungsten was also examined. Figure 14 shows such an interlayer with 50/50 composition, introduced between the steel and tungsten pellets and sintered at 1100 °C. In the composite interlayer, signs of interdiffusion are again visible, but the intermetallic appeared in much smaller amounts than in previous cases at the same temperature and formed rather small spots instead of contiguous layers. Visually good bonding (free of gaps or cracks) of the interlayer to both the tungsten and steel pellets can be seen; only at the steel-composite interface, small pores can be found.

**Figure 15:**
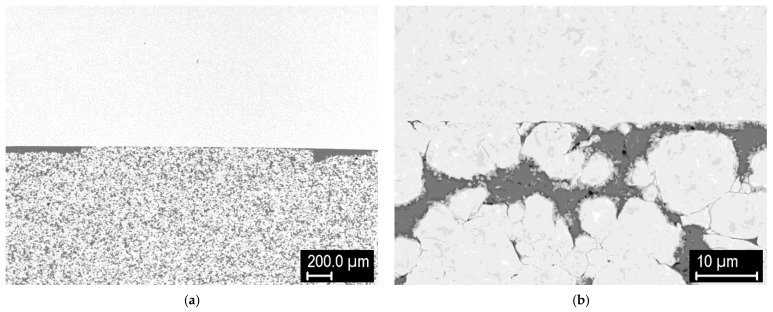
Tungsten-steel joint with a 60/40 mixed and lightly milled interlayer, joined at 1100 °C. (**a**) overview; (**b**) detail of the tungsten-composite interface.

Another example of a composite interlayer is displayed in Figure 15. It consists of 60/40 ratio of tungsten and steel powders, lightly ball-milled, and also with a thin layer of pure steel powder introduced at the interface with tungsten. By comparison of Figure 14a and Figure 15a, one can see that the moderate milling improved the homogeneity of the distribution of the individual phases in the composite. However, the thin layer of pure steel powder did not maintain a uniform thickness upon sintering. The composite again exhibits good bonding with pure tungsten and minor intermetallic formation at the interfaces.

**Figure 16:**
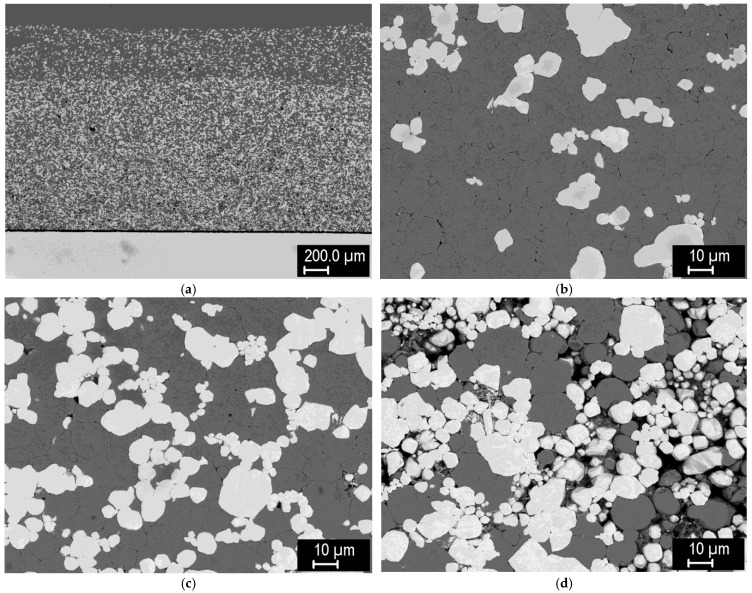
Tungsten pellet with a 4-layer FGM, sintered at 1000 °C. (**a**) overview; (**b**) detail of 20/80 layer; (**c**) detail of 40/60 layer; (**d**) detail of 60/40 layer.

Next, stepwise FGMs of several layers were produced. Figure 16 shows a tungsten pellet with a 4-layer FGM, consisting of 60%, 40%, and 20% W and 100% steel layers (from powders of −20 μm size). This FGM was sintered at 1000 °C in an attempt to avoid the intermetallic formation. While the intermetallic did not form, as expected (Figure 16b–d), the W-rich layer sintered only partially and did not form a proper bond with the pure tungsten.

**Figure 17:**
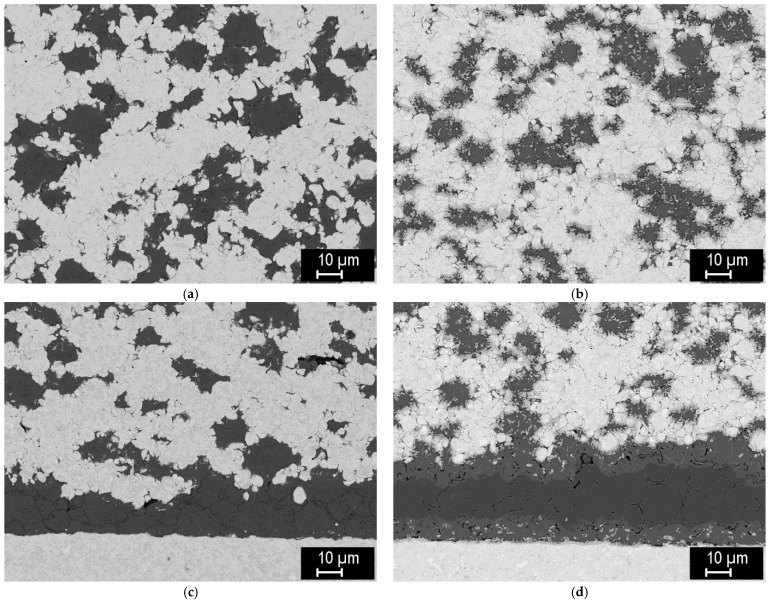
Tungsten pellet with a 60/40 mixed layer with finer tungsten powder. (**a**) Detail of the composite layer, sintered at 1000 °C; (**b**) detail of the composite layer, sintered at 1100 °C; (**c**) detail of the composite-tungsten interface, sintered at 1000 °C; (**d**) detail of the composite-tungsten interface, sintered at 1100 °C.

Figure 17 shows the microstructures of mixed layers (joined again to a tungsten pellet) composed of steel (−20 μm) and tungsten (~4 μm) powders, lightly milled, sintered at 1000 °C (Figure 17a,c) and 1100 °C (Figure 17b,d). Fine tungsten powder was used to see whether the smaller powder size would improve the sintering at a lower temperature in combination with steel. Details of the mixed layers show that the combination of finer tungsten and coarser steel powders resulted in reduced homogeneity of the layer, despite the milling step prior to sintering. Apparently, the finer tungsten particles tend to agglomerate with each other. Localized regions with larger W clusters experienced incomplete sintering (Figure 17d). At 1000 °C, the intermetallic formation was again avoided (Figure 17a), but it formed in significant amounts at 1100 °C (Figure 17b). In both cases, good bonding to the tungsten pellet was achieved (again with a thin interlayer of pure steel powder (Figure 17c,d).

**Figure 18:**
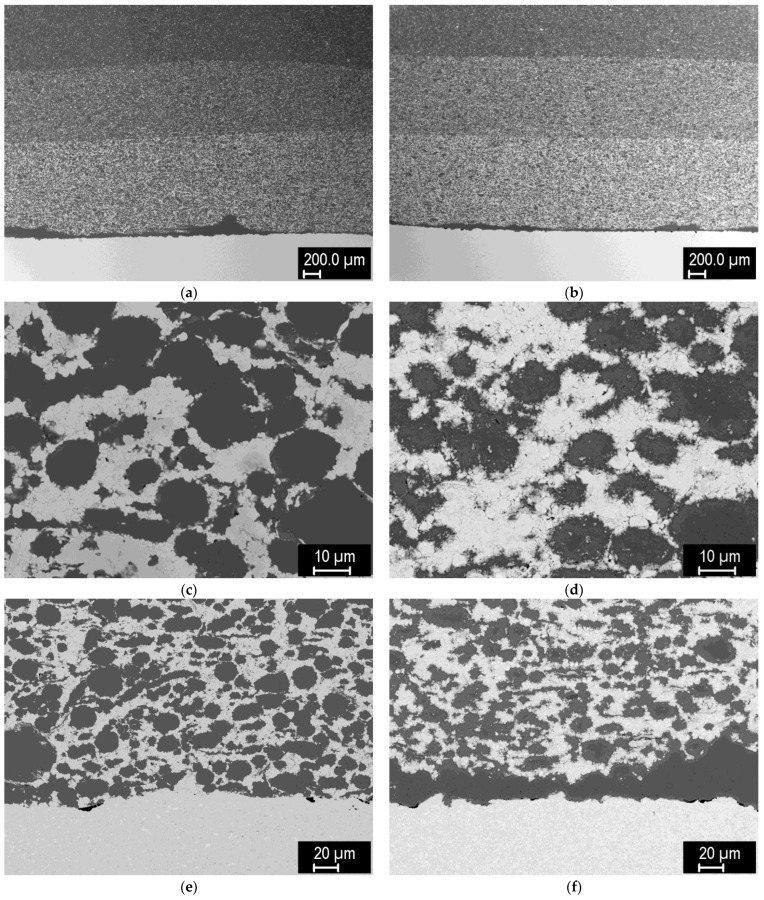
Tungsten pellet with a 3-layer FGM (60/40, 40/60 and 20/80 mixed layers with finer tungsten powder and extended milling. (**a**) Overview, sintered at 1000 °C; (**b**) overview, sintered at 1100 °C; (**c**) detail of the composite layer, sintered at 1000 °C; (**d**) detail of the composite layer, sintered at 1100 °C; (**e**) detail of the composite-tungsten interface, sintered at 1000 °C; (**f**) detail of the composite-tungsten interface, sintered at 1100 °C.

Figure 18 shows tungsten pellets with 3-layer FGMs consisting of 60/40, 40/60, and 20/80 layers of tungsten (4 μm) and steel (−20 μm) powders. This time, the powders were milled more intensely to improve their mixing. As can be seen, the homogeneity of the mixtures is significantly improved compared to the previous case. The composites feature high density, although in the tungsten regions—formed from numerous fine particles of the starting powder—traces of the original particle boundaries can be seen. As expected from the previous samples, the intermetallic phase formed in small amounts at 1100 °C, but did not form at 1000 °C. Good bonding to the tungsten pellet was again observed.

A few concluding remarks regarding the Fe_7_W_6_ intermetallic may be made here. Based on the above results, the ‘boundary’ temperature for its formation appears to be between 1000 and 1100 °C, although local differences in its thickness may occur due to variations in local temperature and current density. Based on the results shown in Figure 18, which could be regarded as an ‘optimized’ case of FGM formation, it could be avoided by using the lower temperature, which, on the other hand, results in incomplete sintering of the tungsten grains. Whether this could be a viable compromise is to be determined by a performance test, such as heat flux testing of complete joints with the FGM interlayers. It might be also noted that although the intermetallic can be avoided at the production step, it is likely to form during service if the composites experience thermal exposure at levels above ~1000–1100 °C. 

## 4. Conclusions

Tungsten, steel, and tungsten-steel composites powders were produced by SPS at a variety of conditions and their basic properties were characterized. Processing windows for pure tungsten and steel were first explored, focusing mainly on the sintering temperature and powder size. For tungsten, fine-grained structures (of the order of μm) were obtained. With decreasing powder size, the temperature needed for full sintering decreases; down to about 1500 °C for the finest W powder. For fully sintered compacts, the hardness (and thus yield strength) and thermal diffusivity increase with decreasing powder size. For steel, a similar influence of the sintering temperature was observed. Tungsten-steel composites were also formed at several compositions and sintering temperatures. At temperatures suitable for proper sintering, i.e., at 1100 °C and above, an intermetallic phase forms due to mutual interdiffusion. Despite its brittle nature, it does not prevent the strength of the composites from increasing with the sintering temperature. At intermediate conditions leading to good sintering yet minimized intermetallic formation, the successful production of FGMs of several layers with discrete compositions and joints with bulk materials was demonstrated. The results can serve as a basis for the processing of tungsten-steel composites and FGMs for plasma-facing components.

## Figures and Tables

**Table 1:** Effect of sintering pressure on porosity (at 1600 °C).

Powder Size (μm)	Pressure (MPa)	Porosity (AM) (%)	Porosity (IA) (%)
2	80	12.21	0.69
4	80	17.32	4.22
2	60	12.31	0.14
4	60	14.75	7.22

AM = Archimedean method on the entire sample, IA = image analysis in the central region. Typical coefficient of variation for the Archimedean method is about 4%; for the image analysis, it ranged from ~10% for porous samples to ~30% for highly dense samples.

**Table 2:** Vickers microhardness of selected tungsten samples.

Powder Size (μm)	Temperature (°C)	HV0.3
−20	1800	232 ± 6
−20	1900	252 ± 12
−20	2000	284 ± 5
−20	2100	348 ± 9
2	1800	364 ± 13
2	2000	373 ± 11
0.7	1600	419 ± 12
0.7	1800	409 ± 15

## Data Availability

Not applicable.
